# Structure-guided design and functional characterization of an artificial red light–regulated guanylate/adenylate cyclase for optogenetic applications

**DOI:** 10.1074/jbc.RA118.003069

**Published:** 2018-04-25

**Authors:** Stefan Etzl, Robert Lindner, Matthew D. Nelson, Andreas Winkler

**Affiliations:** From the ‡ Institute of Biochemistry, Graz University of Technology, Petersgasse 12/II, 8010 Graz, Austria,; the § Max Planck Institute for Medical Research, Jahnstrasse 29, Heidelberg 69120, Germany, and; the ¶ Department of Biology, Saint Joseph's University, Philadelphia, Pennsylvania 19131

**Keywords:** adenylate cyclase (adenylyl cyclase), guanylate cyclase (guanylyl cyclase), photoreceptor, optogenetics, hydrogen-deuterium exchange, allosteric regulation, adenylyl cyclase, bacteriophytochrome, light regulation, photosensor

## Abstract

Genetically targeting biological systems to control cellular processes with light is the concept of optogenetics. Despite impressive developments in this field, underlying molecular mechanisms of signal transduction of the employed photoreceptor modules are frequently not sufficiently understood to rationally design new optogenetic tools. Here, we investigate the requirements for functional coupling of red light–sensing phytochromes with non-natural enzymatic effectors by creating a series of constructs featuring the *Deinococcus radiodurans* bacteriophytochrome linked to a *Synechocystis* guanylate/adenylate cyclase. Incorporating characteristic structural elements important for cyclase regulation in our designs, we identified several red light–regulated fusions with promising properties. We provide details of one light-activated construct with low dark-state activity and high dynamic range that outperforms previous optogenetic tools *in vitro* and expands our *in vivo* toolkit, as demonstrated by manipulation of *Caenorhabditis elegans* locomotor activity. The full-length crystal structure of this phytochrome-linked cyclase revealed molecular details of photoreceptor–effector coupling, highlighting the importance of the regulatory cyclase element. Analysis of conformational dynamics by hydrogen–deuterium exchange in different functional states enriched our understanding of phytochrome signaling and signal integration by effectors. We found that light-induced conformational changes in the phytochrome destabilize the coiled-coil sensor–effector linker, which releases the cyclase regulatory element from an inhibited conformation, increasing cyclase activity of this artificial system. Future designs of optogenetic functionalities may benefit from our work, indicating that rational considerations for the effector improve the rate of success of initial designs to obtain optogenetic tools with superior properties.

## Introduction

Using light to control biological functionalities had already been anticipated as a powerful tool in the late 1970s by Francis Crick. However, at that time scientists were lacking the systems to render single molecules or even cells light responsive. This changed abruptly when the field of optogenetics was created by showing that channelrhodopsins can be used to stimulate cells by light with high spatial and temporal control. Eventually, the impressive advances in the field of neurobiology incited additional efforts to use light-regulatable tools for a variety of biological questions. Over the last decade this enabled unprecedented possibilities in cell biology (reviewed in Ref. 1) and impressive applications from basic research to behavioral studies (2, 3). However, the rational design of systems aiming at the direct control of enzymatic functionalities still proves to be challenging due to our limited understanding of mechanistic details involved in photosensor–effector communication.

To improve our understanding of molecular concepts involved in red light regulation of enzymatic functionalities and to extend the optogenetic toolbox, we focused on an artificial system that links the photosensory domain of a red light–sensing bacteriophytochrome from *Deinococcus radiodurans* (*Dr*BphP, (4)) to a guanylate cyclase (GC)^4^ domain from *Synechocystis* sp. (Cya2 (5)), resulting in a novel red light–controllable GC functionality. Because this cyclase's substrate preference can be easily switched by a single amino acid exchange in the active site (6), our efforts also provide complementary adenylate cyclase (AC) tools.

Previously, red light–regulated ACs had been successfully generated by directly fusing the cyclase core domain of CyaB1 from *Nostoc* sp. to the C-terminal helix of the phytochrome sensory module of BphG1 from *Rhodobacter sphaeroides* (7). Although the initial photodynamic range was relatively small (2-fold), the system could be further improved by random mutagenesis and considerable screening efforts to a state where it can be applied to manipulate thrashing behavior in *Caenorhabditis elegans*. Because residual dark activity of the activatable cyclase (termed IlaC) was attenuating the dynamic range of the optogenetic tool, we wanted to employ a different strategy in fusing sensor and effector domains that would allow us to reduce dark-state activity and to improve the dynamic range. Based on available crystal structures of blue light–activated cyclases (8, 9) and human soluble AC (10–14), we decided to include a conserved structural feature N-terminal to the cyclase core in our new fusions. This structural element can be found in many natural class III cyclases and it has recently been described as cyclase transducer element (CTE), indicating its importance for the regulation of cyclase activity (15). Because both phytochromes and class III cyclases exist as functional dimers and both the C-terminal end of the phytochrome photosensory module and the N-terminal part of the CTE adopt α-helical structures, we anticipated that finding an appropriate fusion point in the resulting coiled-coil structure should provide novel light–regulated systems with improved properties compared with previous designs (7). Based on suggested mechanisms of signal transduction in related coiled-coil containing systems (8, 9, 16–18), the degree of coiled-coil character and the overall length of the helical linker spanning the fusion point were considered as central parameters for tuning the functional coupling of sensor and effector. Starting from an initial construct we created a series of variants with sequential single amino acid truncations and elongations of the phytochrome part at the sensor–effector fusion point. The resulting constructs (phytochrome-activated guanylate cyclases (PagCs) or phytochrome-activated adenylate cyclases (PaaCs) for constructs based on the ^GC^E488K substitution (6)) sample different linker lengths and relative positions of the cyclase dimer with respect to the phytochrome over more than two full coiled-coil heptad repeats.

We identified three light–regulated constructs, two featuring a strong light activation and one being inversely regulated. With dynamic ranges being higher than ∼5-fold for all these constructs, this demonstrates that a more rational approach, which includes functionally important elements of the regulated enzymatic functionality, has the ability to improve the initial design of novel optogenetic tools. To understand the molecular mechanism of such optogenetic tools, we solved the dark-state crystal structure of one light–activated construct revealing molecular details of an artificial full-length optogenetic tool based on a red light–sensing phytochrome. This structure of *D. radiodurans* bacteriophytochrome with its PAS-GAF-PHY photosensory module (PSM) fused to a non-natural enzymatic output domain shows interesting details about the functionally important linker region between sensor and effector and adds a new, potentially inactive, conformation to the currently emerging structural details of CTEs. Together with analyses of the changes in dynamics upon light illumination, this provides important insights into the functioning of an optogenetic tool that will be helpful for the future design and optimization of other optogenetic systems.

## Results

### Design of red light–regulated cyclases

**Figure 1.**
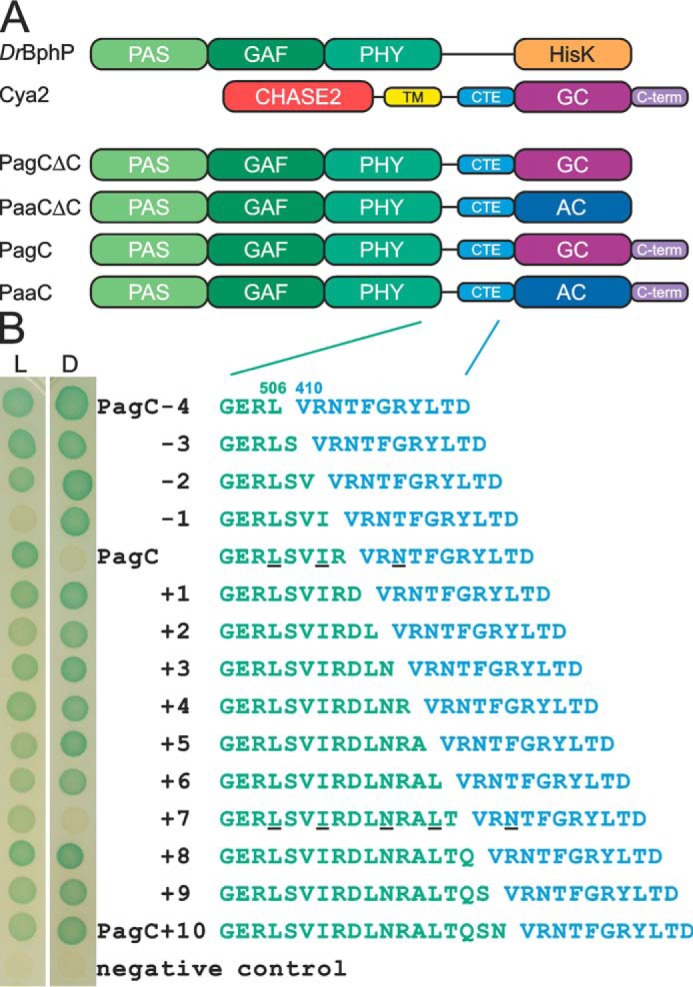
**Domain architecture and construct design.** *A,* domain architectures of the *Dr*BphP bacteriophytochrome (*PAS-GAF-PHY*) with the natural histidine kinase effector (*HisK*) and the guanylate cyclase Cya2 (GC), which is attached to a light–independent CHASE2 extracellular sensory domain, separated by a transmembrane domain (*TM*). The cyclase core is N terminally preceded by a CTE. The C terminus is explicitly shown to point out the difference between the initial series of constructs featuring the phytochrome fused to the C terminally-truncated cyclase (PagCΔC/PaaCΔC) and the final series including the native C terminus (PagC/PaaC). PaaCΔC and PaaC are fused to a Cya2 E488K variant exhibiting AC activity. *B, left*: screening of PaaC constructs in cyclase-deficient *E. coli* on LB agar supplemented with X-Gal under red light illumination (*L*) and in the dark (*D*). *Green* coloration of the colonies indicates adenylate cyclase activity. *Right*: amino acid sequence spanning the fusion point between phytochrome (*green*) and cyclase (*blue*) of each of the tested constructs. *Underlined* residues highlight the generally hydrophobic *a* and *d* positions in the (*abcdefg*)-heptad coiled-coil repeats.

A series of 15 chimeric proteins featuring the PSM of *D. radiodurans* bacteriophytochrome (residues ^PSM^1–506 to 520) fused to the cyclase core domain of the originally CHASE2-regulated (19) Cya2 from *Synechocystis* PCC6803 (residues ^GC^410–638, the uppercase GC indicating a numbering scheme following previously published reports of the effector domain (6)) was screened for red light-dependent adenylate cyclase activity in adenylate cyclase-deficient *Escherichia coli* BL21(DE3). Due to the cAMP-based initial screening procedure in *E. coli*, the AC variants (featuring ^GC^E488K) were employed. Because these initial constructs (PaaCΔCs) turned out to have rather low activities, we decided to include the native C-terminal extension of the cyclase domain for all subsequent designs (residues ^GC^410–756, Fig. 1*A*). These constructs featured substantially increased AC activity and we observed light regulation in three cases (Fig. 1*B*). Two constructs showed low dark-state activities and a pronounced increase upon illumination (PaaC, PaaC+7), whereas one other construct was active in the dark, but showed substantially impaired activity upon illumination (PaaC-1). Interestingly, all other constructs feature a similar basal activity under both dark conditions and with continuous illumination. The three promising PaaC constructs from the initial screening, as well as their PagC counterparts, were then expressed, purified, and biochemically characterized.

### Spectroscopic characterization

**Figure 2.**
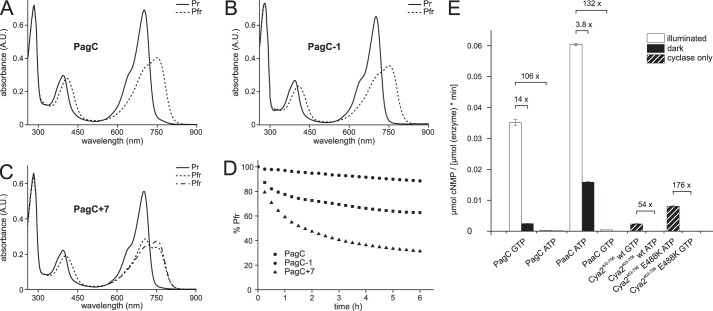
**UV-visible spectra of light–regulated constructs and activity plots of PagC constructs.** *A–C,* UV-visible absorption spectra of PagC, PagC-1, and PagC+7, respectively. *Full lines* show Pr state spectra, *dashed lines* correspond to the Pfr spectra. The *dot-dashed trace* for PagC+7 shows a Pfr spectrum from 500 to 900 nm that was recorded in the presence of constant 660 nm illumination with a CCD-based spectrophotometer to minimize thermal reversion to Pr during data acquisition. *D,* dark-state recoveries of the three constructs indicated in % Pfr over the course of 6 h. *E,* enzymatic activities of PagC, PaaC, and cyclase-only variants. *White bars* indicate light-state activity and *black bars* show the activity in the dark-state. *Hatched bars* correspond to the cyclase-only variants. *Brackets above* the bars show the fold-difference between light/dark activities as well as an indication for the substrate specificity. Initial rates were measured at 1 mm GTP/ATP for different time points in a time frame where overall substrate conversion was below 10%. *Error bars* show the error of the estimate for linear fits of cNMP production weighted by the reciprocal of the square of the standard deviation from three experimental replicates.

All constructs were successfully expressed with high yields, however, even in the presence of coexpressed heme oxygenase, the soluble fraction required supplementation of biliverdin during purification to quantitatively obtain the holoproteins. The initial biochemical characterization by size exclusion chromatography indicated that the proteins form stable dimers in solution, both in the dark and under constant red light illumination. UV-visible absorption spectra of all three PagC proteins show the characteristics of the *Dr*BphP phytochrome (Fig. 2, *A–C*). Red light illumination converts the proteins form the dark-adapted red light–absorbing state (Pr) to the far red–shifted state (Pfr). The thermal dark reversion rates back to the Pr state differ substantially among the three fusion constructs (Fig. 2*D*).

### Activity assays

Because light–inducible cyclase functionalities are tentatively more interesting for optogenetic applications and because the dynamic range of the PaaC construct was largest in the screening (Fig. 1*B*), we subsequently focused on the detailed characterization of the full-length PaaC and PagC constructs (DrBphP^1–510^–Cya2^410–756^). Analysis of steady-state kinetics showed subtle differences with respect to the cooperativity of nucleotide conversion and the magnitude of substrate inhibition for the ATP- and GTP-specific constructs (Fig. S1 and Table S1). Comparison of activities close to the observed activity maximum at 1 mm GTP revealed a 14-fold light activation for PagC (Fig. 2*E*). The PaaC variant is slightly more active than PagC when comparing the respective dark and light conditions; however, the dynamic range is reduced to ∼4-fold at 1 mm ATP. Both PagC and PaaC are specific for their corresponding substrate as they show only minor conversion of the respective other nucleotide. Interestingly, the cyclase constructs without the PSM (Cya2^403–756^ WT and ^GC^E488K), which also elute as dimers from the size exclusion column, are less active than the sensor–effector fusions. However, the specificity as well as the higher activity of the AC variant compared with GC WT are similar (Fig. 2*E*). In line with the results from the initial screening efforts, the *in vitro* activities of the purified PaaCΔC and PagCΔC versions for cNMP formation are very low and in most cases below the detection limit. In the case of PaaCΔC, removal of the C terminus appears to favor side reactions that result in minor amounts of various dephosphorylation products, such as ADP, AMP, and adenosine.

### Crystal structure of a dark-state bacteriophytochrome–linked nucleotide cyclase

**Figure 3.**
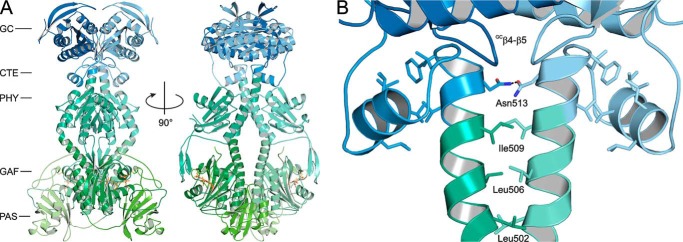
**Structure of PaaCΔC in the Pr state.** *A,* overview of the crystal structure of PaaCΔC in cartoon representation with individual domains colored according to the scheme in Fig. 1*A*. The biliverdin chromophores (*orange*) and their covalent attachment sites (Cys-24) are shown in *stick* representation. *B,* close-up view of the linker-CTE region with residues involved in coiled-coil formation and in packing of the short CTE helix highlighted in *stick* representation.

To better understand molecular details of light activation in these phytochrome-linked cyclases, we attempted crystallization of both PagC and PaaC full-length variants in the presence or absence of substrates, as well as under dark and light conditions. However, no crystals could be obtained for the constructs in the presence of the 118-amino acid C-terminal extension. Even though the extension is important for stimulating cyclase activity, we attempted crystallization of the truncated ΔC constructs because the core cyclase also features AC/GC activity (Fig. 2*E*) (6). We eventually obtained crystals grown under dark conditions that allowed us to solve the crystal structure of the full-length photosensory module linked to the cyclase effector in the dark-state conformation to a resolution of 2.35 Å (Fig. 3*A*). The best diffracting crystal was obtained from the ATP-specific variant, but also PagCΔC forms identical crystals under the same conditions. Due to the almost identical crystal structure of the PagCΔC construct compared with the deposited PaaCΔC structure, we do not expect differences in the overall mechanism of signal transduction and regulation.

**Figure 4.**
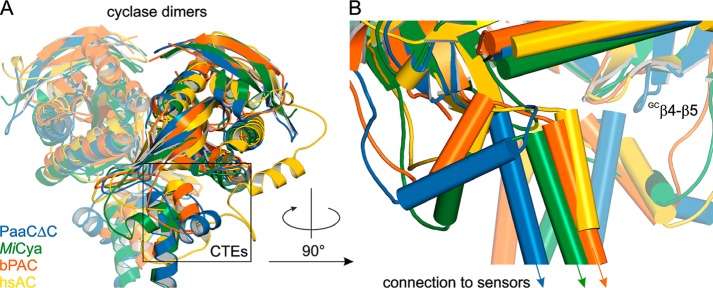
**Comparison of the PaaCΔC cyclase domain to other cyclase structures.** *A,* superposition of various cyclase structures crystallized with their respective CTEs shows that the cyclase core dimer in PaaCΔC aligns well with all other structures (*blue,* PaaCΔC; *yellow*, PDB 4CLF (human soluble AC); *orange*, PDB 5M2A (bPAC from *Beggiatoa sp.*); *green,* PDB 5O5K (Cya from *Mycobacterium intracellulare*)). Structures were aligned by superposition of all chains “A” and 4CLF residues 1–263. *B,* close-up of the linker-CTE region from the cyclase structures shown in *A*. Unlike in other cyclases, the short CTE helix is “folded back” and residues in the loop preceding the short helix interact with the ^GC^β4–β5 tongue, which is displaced compared with other structures. 4CLF residues 236–262 are not displayed and clipping planes have been adjusted for clarity.

The structure of PaaCΔC consists of two chains in the asymmetric unit and shows a dimeric arrangement of the protein in its Pr state, indicated by the 15*Z*-conformation of the biliverdin chromophores (Fig. S2) and the typical β-hairpin structure of the PHY domains' tongue elements (20, 21). The overall dimer is almost perfectly symmetric; however, several differences between the two chains exist on specific structural elements throughout the dimeric assembly. The loop between β3 and β4 (residues ^PSM^104–107) on the outside of the PAS-GAF bidomain show different conformations in both chains, most probably due to crystal contacts. In addition, there is considerable asymmetry in the loop connecting β5 of the PAS domain and α4 of the GAF domain (residues ^PSM^130–138), as well as for the side chains of ^PSM^Tyr-307. This is interesting, because both the β5–α4 loop and ^PSM^Tyr-307 are situated at the dimer interface and in most other PSM structures of *Dr*BPhP no density can be observed for β5–α4 loop. Interestingly, the only other structures that also show a defined electron density at the above mentioned interface region are the activated Pfr structures of the *Dr*BphP PSM, which also feature a repositioning of the PHY domain relative to the PAS-GAF module (21, 22). The overall dimer arrangement of the PAS-GAF domains is, however, identical to other phytochrome structures and only subtle differences in the PHY domains' overall positioning can be observed. As expected from the periodicity of hydrophobic residues in the linker element of the PagC fusion between the PHY domain and the CTE, the linker helices adopt a characteristic coiled-coil arrangement (Fig. 3*B*). This results in a tighter packing of the PHY domains and the presence of a stronger kink in the connecting helices between GAF and PHY domains when compared with currently available *Dr*BphP PSM-only structures (20, 21). However, the overall structure of the PHY domains is not affected by the presence of an output domain. The start of the coiled-coil linker follows the two glycine residues present in the terminal phytochrome helix and is indicated by a kink of the helices where the side chains of ^PSM^Leu-502 interact. Coiled-coil characteristics are maintained throughout the helices, spanning the fusion point until ^GC^Asn-412 (absolute numbering Asn-513). Interestingly, residues at the C-terminal end of the linker helices of this light–activated construct are not in the coiled-coil register that would be expected based on CTE conformations of other class III cyclase structures (9, 12, 23). The constructs from the PagC series corresponding to the CTE in an identical helical register to previously observed transducer element conformations (PagC+1 and PagC+8; with the conserved CTE motif -FGRY- (15) starting at position *d* of a heptad repeat) did not show light regulation in the screenings. Although the overall cyclase dimer closely resembles those of other known cyclase structures (Fig. 4*A*) (9, 12, 23) as well as the isolated Cya2 structure (6), residues in the short CTE helices do not interact with residues from the other protomer as observed in all other CTE structures. They rather interact with the linker helix of the same protomer, thus “folding back” before proceeding to the cyclase core domain (Fig. 4*B*, see also Fig. 3*B*). Side chains from the loop between the linker helix and the short CTE helix cause a displacement of a functionally relevant β-hairpin structure of the cyclase (^GC^β4–β5 tongue), which was shown to play an important role in quaternary structure formation and positioning of catalytic residues in a blue light–regulated cyclase (8, 9). Interestingly, the side chains of the conserved ^GC^Arg-416 of the CTE motif at the C-terminal end of the coiled-coil linker point toward the interface between the cyclase monomers, positioning the guanidinium part in the center of the cyclase dimer below the substrate-binding region of the active site. In the crystal structure the side chains of ^GC^Arg-416 coordinate a sulfate ion due to the presence of high concentrations of ammonium sulfate in the crystallization conditions, with a certain degree of asymmetry between the two chains in this region. An additional structure from a PagCΔC crystal grown in the absence of sulfate ions shows a similar side chain arrangement, although the asymmetry between the two chains is not as pronounced.

The side chains of the residue determining the base specificity (^GC^Lys-488) are not well-defined in the electron density of both protomers in the absence of substrate molecules. Otherwise, the overall cyclase structure is almost identical to the core guanylate cyclase dimer reported previously (6). Therefore, we do not expect pronounced structural rearrangements between the AC-specific PaaC variant and its GC counterpart. Regulation of cyclase activity is most likely achieved by the modulation of the conformational dynamics of functionally important structural elements, such as the ^GC^β4–β5 tongue (9), and thereby modulates the reactivity of the cyclase dimer independent of specific residues involved in the reaction mechanism at the active site.

### Conformational dynamics of PagCΔC (HDX-MS)

**Figure 5.**
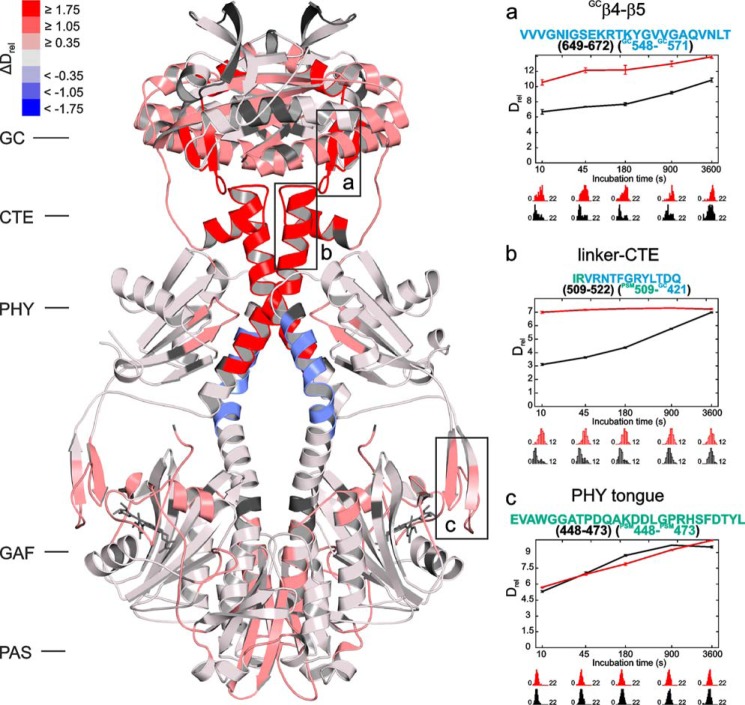
**HDX-MS analysis of PagCΔC reveals changes in conformational dynamics upon red light illumination.** *Left,* PaaCΔC structure colored according to the differences in deuterium incorporation of ΔDrel upon red light illumination (Drel, *light*; Drel, *dark*) after 10 s of deuterium exchange. *Red* coloration indicates an increased, *blue* a reduced deuterium incorporation, respectively, as indicated by the *scale bar at the top left. Right,* relative deuterium uptake (D_rel_) of representative peptides from the ^GC^β4–β5 tongue (*a*), the linker helix spanning the fusion point between PHY domains and CTE (*b*), and the PHY tongue region (*c*). The corresponding sequences are shown on *top* of the plots with absolute and construct-specific numbering. Data are shown as the mean of three independent measurements and *error bars* correspond to the standard deviation. The *lower parts* depict abundance distributions of deuterated species ranging from undeuterated to fully exchanged amide protons in the respective peptides.

To further characterize the role of certain structural elements in intramolecular signal transduction, we analyzed the conformational dynamics of PagCΔC in the dark and under constant red light illumination by hydrogen–deuterium exchange coupled to MS (HDX-MS) experiments (Fig. 5 and Figs. S3 and S4). The analysis of HDX-MS data revealed an increase in conformational dynamics upon illumination around the biliverdin-binding pocket and at the interface between the GAF domains, as well as in the PHY tongue region. However, at intermediate exchange time points, the PHY tongue shows less deuterium exchange in the light than in the dark, indicating a relative stabilization (Fig. 5*c*). A detailed functional interpretation of this exchange pattern, however, is complicated due to the length of the peptides and the pronounced structural rearrangements from β-hairpin to α-helix described for this region (21, 22). In contrast to the relatively dynamic tongue element, the central helices connecting the GAF and PHY domains at the dimer interface show substantially slower deuterium exchange kinetics. Interestingly, at the earliest exchange time point, the region closer to the PHY domains shows reduced deuterium incorporation in the illuminated state, indicating a relative stabilization upon light activation. The core PHY domain does not show any strong light–induced changes in dynamics; however, the helices following the tongue elements and protruding toward the CTE region show pronounced destabilization for all time points. The rate of deuterium incorporation in the respective peptides (Fig. 5*b*) suggests large secondary structure rearrangements up to a complete loss of ordered structure upon illumination. The ^GC^β4–β5 tongue, which is positioned in direct contact with the CTEs, also shows increased dynamics in the light state (Fig. 5*a*). The change of deuterium incorporation in this region can be narrowed down to a few residues in the loop between ^GC^β4 and ^GC^β5 by comparison of the exchange behavior of overlapping peptides (*cf*. Fig. S3). The interaction of this loop with the CTE was also identified as a critical regulatory interaction in the family of blue light–regulated adenylate cyclases (8, 9).

To further address the role of the structurally uncharacterized Cya2 C-terminal extension we performed HDX-MS investigations on the full-length proteins (PagC and PagC-1). Although the local changes in conformational dynamics remain the same between the truncated ΔC variants and the full-length proteins, the magnitude of these changes differs in various regions, including the sensor–effector linker region (Fig. S5). A more detailed characterization of the role of the C terminus is subject to ongoing studies.

### PaaC performs similarly to IlaC22 k27 during in vivo manipulation of locomotor behavior

**Figure 6.**
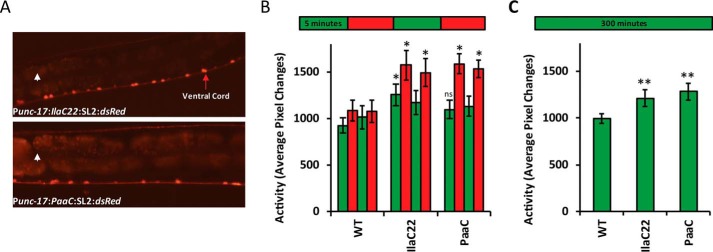
**PaaC application *in vivo* is comparable with IlaC22 k27 but has slightly reduced “dark-state” activity.** *A,* two independent lines each were created where IlaC22 k27 or PaaC were expressed in cholinergic motor neurons (*red arrow* indicates the ventral cord; *white arrows* indicate the autofluorescence of the intestines). *B,* behavioral analyses of worm activity were performed using the WorMotel system. Activity was quantified during illumination with nonactinic green light and activating red light where the light regime consisted of 5 min of green light followed by 5 min of red light in two rounds. WT worms did not respond significantly to the altering light conditions, whereas the transgenic lines expressing IlaC22 k27 or PaaC were significantly more active under red light. Additionally, the IlaC22 k27-expressing strains but not the PaaC-expressing strains were significantly more active under the initial green light exposure compared with WT animals (*, *p* < 0.05, WT *n* = 108, IlaC22 k27 *n* = 116, PaaC *n* = 216 animals, 18 trials). A time lapse movie of frames from 10-s intervals is provided in Movie S1. *C*, to measure dark-state activation we quantified locomotor activity under green light for an extended period of time (300 min) and found that both IlaC22 k27- and PaaC-expressing strains were more active than WT and not significantly different from one another (**, *p* < 0.001, WT, *n* = 18; IlaC22 k27, *n* = 18, PaaC *n* = 32 animals, 3 trials).

To test how the improved dynamic range and the reduced dark-state activity translate into an *in vivo* system, we performed a direct comparison to a previously established red light–activated AC (7) in the animal model system *C. elegans*. To this end, we generated transgenic animals expressing either PaaC or the previously characterized IlaC22 k27 in cholinergic motor neurons (Promoter: *unc-17*). The *unc-17* promoter drove expression of an operon that contained the *C. elegans* codon-adapted sequence of either enzyme and the coding sequence for dsRED, to verify the location of expression. Two independent lines for both IlaC22 k27 and PaaC were created and the expression pattern was verified (Fig. 6*A*). To measure locomotor activity we used the WorMotel system (24) to quantify the average pixel changes between frames obtained every 10 s of each recording (Movie S1). First, to determine *in vivo* functionality of PaaC and to compare directly to IlaC22 k27, we measured locomotor activity during 5-min intervals of the following pattern of light exposures: green, red, green, red (Fig. 6*B*). Whereas control animals did not show significant stimulation by red light, both IlaC22 k27 and PaaC featured a pronounced increase in activity. Although the maximum activity under red light conditions of both optogenetic tools was comparable, the green light (*i.e.* dark state) activity of PaaC was slightly lower than that of IlaC22 k27. Although IlaC22 k27 activity under green light was significantly higher than that of WT, PaaC was not. However, the difference between IlaC22 k27 and PaaC did not reach the level of significance (Fig. 6*B*). Because the IlaC22 k27 strain features a significantly elevated resting state activity compared with WT worms, these results suggested that the *in vivo* dynamic range of the PaaC tool might be better than that of IlaC22 k27 under the conditions of this assay. To address this further, we measured average activity under constant green light for an extended period of time (5 h) and found that the green light activity was significantly higher for both IlaC22 k27 and PaaC compared with WT, but not significantly different from one another (Fig. 6*C*). This suggests that green light stimulates PaaC to levels similar to that of IlaC22 k27 over time. Thus, if experiments are designed appropriately, where any light exposure is limited, PaaC is the better option to allow for an enhanced dynamic range. It should also be noted that, expression levels were higher for the PaaC-expressing strains based on the intensity of the dsRED fluorescence, thus, for future experiments, expression levels might be an important additional parameter during the experimental design process. Considering functionally important elements in the initial stages of optogenetic tool design, therefore has enabled the rational improvement of an optogenetic system by decreasing its dark-state activity and providing a higher dynamic range; not only *in vitro* but potentially in experiments with an animal model system.

## Discussion

The bacteriophytochrome from *D. radiodurans* is one of the best studied representatives of red-light sensing photoreceptors, yet there is little structural information as to how sensor and effector are functionally coupled to enable intramolecular signal transduction upon light activation. Recent crystal structures of the PSM module of *Dr*BphP in its Pfr state suggested pronounced conformational changes upon illumination due to an opening of the PHY domains (21, 22), however, in the context of the full-length protein more subtle changes were shown to be sufficient to communicate the light signal from the sensory module to the enzymatic effector (25). Small scale rotational and/or translational rearrangements of the PHY domains are also in line with mechanisms of regulation observed in histidine kinases, where small conformational changes allow switching between active and inactive states (26). Similar subtle structural rearrangements have also been postulated for the activation of phytochrome-linked diguanylate cyclases, where light activation results in a population shift of different coiled-coil registers in the sensor–effector linker (27).

Analysis of our artificial phytochrome-linked cyclase construct provides further insights into the molecular functioning of phytochromes coupled to an output domain. Results from HDX-MS measurements for PagCΔC support a mechanism of release from a dark-state inhibited conformation by rearrangement of the PHY domains' tongue elements that induces changes in conformational dynamics at the dimer interface. The length of peptides covering the tongue region and their complex exchange behavior prevent a detailed molecular interpretation of the light–induced conformational changes. Because others have conclusively shown that light activation of *Dr*BphP is accompanied by refolding of the tongue element into an α-helix (21, 22), we believe that this extensive structural rearrangement that is also accompanied by loss of ordered structure is in line with the complex observations presented here. The increase in dynamics in the tongue at early time points is accompanied by a stabilization of the helices connecting the GAF and PHY domains. The kink in these helices, which is slightly more pronounced in the structure of the PSM-cyclase fusion compared with PSM-only structures of *Dr*BphP, could serve as a hinge for transducing conformational changes to the PHY domains. This would be in accordance with the observation of the pronounced opening of PHY domains in the Pfr state structure of the *Dr*BphP PSM (21, 22) as well as with the compatibility to various output domains that require only subtle rearrangements of the catalytic dimer architecture or its dynamics via the PHY element (*Dr*BphP (25), PagC, and LAPD (18)). Ultimately, the conformational changes in the photoreceptor modules lead to a destabilization of the coiled-coil linker helices. In the particular case of the nonnatural cyclase effector this causes a further destabilization of the CTEs and hence the release of the cyclase dimer from a “locked” unproductive state in the case of our light–activated construct. In the dark state, the CTE helix interacts via hydrophobic packing with the linker helix of the same protomer, which is different from other known class III cyclases, where the CTE helix packs against the linker helix of the other protomer. The loop between the linker helix and CTE helix interacts with the ^GC^β4–β5 tongue of the cyclase, which contains residues that protrude into the substrate-binding area, and might play a functional role in relaying the allosteric signal toward the active sites. Residues in the loop between ^GC^β4 and ^GC^β5 also show increased deuterium incorporation upon illumination, which supports previous observations for a functional role of this element in sensor–effector coupling (8, 9).

Interestingly, the linker-CTE region is also affected by the inclusion of the C-terminal extension (Fig. S5), further supporting the importance of this element in regulating cyclase activity and also indicating one potential mechanism of how the C terminus could stimulate cyclase activity via intra-protein interactions. However, it should be noted that the proposed mechanism of cyclase activation in the artificial PagC/PaaC constructs might differ from naturally occurring cyclases. Especially, because the light–inactivated constructs (PagC-1 and PagCΔC-1) feature a similar CTE destabilization upon illumination (Fig. S5), which appears to shift the cyclase dimer from a productive conformation into a less active state. The effect of signal reversion upon deletion of a single amino acid in the helical linker also has been observed in an artificial blue light–regulated histidine kinase (28). Changes of the helical register by deletions or insertions potentially cause a rotation of the effector domains relative to the phytochrome sensor and hence signal inversion might be a direct consequence of specific structural rearrangements upon light activation of the sensory module that involve subtle differences in rotation or translation of the effector domains. Therefore we anticipate that the relative positioning of sensor and effector domains with respect to the helical register of the coiled-coil linker is an important prerequisite for their functional coupling. This is supported by the observation that both light–activated constructs (PagC/PaaC and PagC+7/PaaC+7) differ by seven amino acid residues (*i.e.* two full helical turns) in linker length and should therefore have very similar structural arrangements of the PSM and the cyclase. This helical periodicity in artificial light–regulated constructs has been observed in similar photoreceptor engineering studies (7, 28). However, the light–inactivated PagC-1/PaaC-1 and the seven-residue longer PagC+6/PaaC+6 constructs do not show the same behavior, indicating that there are other determinants of signal transduction besides the relative positioning of sensor and effector.

It has been shown that coiled-coil structures can be bistable switches, changing their conformation, *i.e.* helical register, by a relative rotation or translation of the helices (17). The equilibrium between these alternate registers depends in principle on the number and quality of coiled-coil interactions in either register and is therefore sequence-dependent. In addition, the relative population of different registers might be influenced by the light–mediated rearrangement of the PHY domains upon release from a restrained dark-state conformation of the PHY tongue element. Depending on the intrinsic stability of the coiled-coil, the PHY domains' rearrangements might stabilize or destabilize a certain register and thus influence the CTE stability and ultimately the cyclase activity. Thus, phytochrome activation results in an increase of conformational freedom of the terminal PHY domains and their dimeric interface extending into the sensor–effector linker element, but how this signal is eventually integrated by the enzymatic effector in terms of (in)activation or dynamic range is mainly dependent on the nature of the linker helices.

Considering the markedly different effects of red light illumination on dynamic properties of functionally important elements of phytochrome signaling between *Dr*BphP and *Is*PadC (27), using different phytochrome sensors in the design of optogenetic tools will obviously result in significantly altered functional characteristics. It is important to note that also the effector domain and the linker region have an influence on the properties of the sensor, as the different Pfr state stabilities of our artificial fusion constructs show. Taking all this into account, phytochrome signaling is a complex process based on the interplay of dynamic equilibria of several structural elements that are involved in signal integration and transduction. In many natural sensor–effector combinations, as well as in successfully engineered fusions, a more or less pronounced coiled-coil character is present in the linker, and clearly the complex interplay of conformational dynamics is one reason why the rational design of new optogenetic tools still remains challenging for each sensor–effector combination.

Nevertheless, including functionally important structural elements in the initial designs of optogenetic tools increases the chances of obtaining promising starting constructs, as in our case a red light–regulated cyclase functionality with a reasonable dynamic range and low dark-state activity. Compared with a previously described red light–regulated adenylate cyclase (IlaC22 k27 (7)), the dark-state activity is significantly reduced *in vitro* (∼50-fold lower). In addition, PagC and PaaC can serve as versatile optogenetic tools, because they can be used for applications targeting cAMP, as well as cGMP-dependent processes. With their low dark-state activity they are optimally suited to increase the repertoire of light–regulated cyclase functionalities if the expression levels can be adjusted experimentally to match the required window of cyclic nucleotide concentration variations *in vivo*. The benefit of the low dark-state activity of the PaaC system was also demonstrated in direct comparison with the IlaC22 k27 tool in *C. elegans*. Although the higher dark-state activity of IlaC22 k27 resulted in elevated locomotor activity under nonactinic light conditions, this stimulation by green light was less pronounced for PaaC during a short exposure. However, during exposure to prolonged periods of green light, transgenic animals expressing either IlaC22 k27 or PaaC were both significantly more active than controls. This suggests that longer exposures to green light stimulate the PaaC enzymes to levels similar to those of IlaC22 k27. However, we also observed qualitative differences in expression levels between the IlaC22 k27- and PaaC-expressing strains; the dsRED fluorescence in the PaaC-expressing lines was more intense. So, it is possible that with higher expression the activity during green light exposure is more pronounced. Thus, to optimize the *in vivo* response, both expression levels and the time of exposure should be considered. A higher dynamic range makes this optimization step easier and increases the applicability of optogenetic tools in general.

## Experimental procedures

### Cloning and transgenic animal construction

An initial synthetic gene construct for a fusion of the bacteriophytochrome from *D. radiodurans* (*Dr*BphP) and the core guanylate cyclase domain from *Synechocystis* sp. (Cya2, E488K variant) was ordered (Life Technologies) and cloned into the pET M11 vector downstream of a TEV-cleaveable His tag using NcoI and NotI restriction sites. Addition of the C-terminal cyclase extension was performed by Gibson assembly using a synthesized GeneStrand (Eurofins) with complementary regions to the pET M11 vector and the 3′-end of the coding region of the cyclase core. DNA assembly was performed using the NEBuilder kit (New England Biolabs) according to the instruction manual. Additional constructs were created by PCR mutagenesis using an adapted QuikChange protocol (29) (primers used in this work are listed in the supporting Information). Resulting plasmids were sequence verified and used for subsequent experiments.

The DNA constructs expressed in *C. elegans* were made by overlap-extension PCR, as previously described (30). Briefly, the *unc-17* promoter, which expresses specifically in cholinergic motor neurons (31), was amplified using PCR from genomic DNA. Next, the coding sequences of either IlaC22 k27 or PaaC were amplified from plasmids: pSJU1 or pMA-T_PaaC, respectively. Both sequences were codon-adapted for enhanced expression in *C. elegans* (32). Last, the operon sequence from the genes *gpd-2*/*-3* and the dsRED coding sequence were amplified from the plasmid pLR304 (a gift from David Raizen). The three pieces were fused together by PCR; the oligos used are listed in the supporting Information. Microinjection of DNA was performed as described (33).

### Screening of cyclase activity in fusion constructs under dark- and red-light conditions

*E. coli* BL21(DE3) cya^−^ cells containing the helper plasmid pT7Ho1 were transformed with plasmids encoding the adenylate cyclase variants. The pT7Ho1 helper plasmid for biliverdin generation is derived from the originally described version but has its kanamycin cassette replaced by a chloramphenicol resistance marker (27, 34). Cells were grown in 30 ml of LB medium supplemented with 0.36% glucose and antibiotics for 6 h at 37 °C. An aliquot was then harvested, the cells were collected by centrifugation and resuspended in LB medium with antibiotics to reach an *A*_600_ of 20. 4 μl of the cell suspension were spotted on LB agar plates (10 μm isopropyl β-d-thiogalactoside, 60 μg/ml of 5-bromo-4-chloro-3-indolyl-β-d-galactopyranoside (X-Gal), 10 μg/ml of 5-aminolevulinic acid, antibiotics) and incubated in the dark or under constant red light illumination for several hours at 30 °C, until a change in coloration of the colonies was observed.

### Protein expression and purification

Plasmids were transformed into chemically competent *E. coli* BL21(DE3) cells carrying the helper plasmid pT7Ho1. Subsequent liquid cultures in LB medium supplemented with 0.36% glucose and antibiotics were grown at 37 °C to mid-logarithmic phase and 5-aminolevulinic acid added to a final concentration of 10 mg/liter. After reduction of the temperature to 16 °C, expression was induced by the addition of 100 μm isopropyl β-d-thiogalactoside. Cells were further incubated at 16 °C and harvested after 16 h. Pellets were stored at −20 °C. For lysis, cells were resuspended in lysis buffer (50 mm Tris (pH 8.0), 300 mm NaCl, 2 mm MgCl_2_, 10 mm imidazole) supplemented with 0.5 mg/ml of lysozyme and protease inhibitor (cOmplete ULTRA Tablets, Roche Applied Science). After sonication (Labsonic L, Sartorius) the lysate was cleared by ultracentrifugation (206,000 × *g*) and the supernatant subjected to a gravity flow nickel-nitrilotriacetic acid (Ni-NTA) affinity column (Ni-Sepharose 6 Fast Flow, GE Healthcare). After a wash step with 10 column volumes of lysis buffer supplemented with 25 mm imidazole, the protein was eluted with 200 mm imidazole in lysis buffer and the elution fractions containing the desired protein were supplemented with an excess of biliverdin. His tags were removed using 1/15 (w/w) in-house expressed TEV protease in an overnight dialysis step at 4 °C in dialysis buffer (50 mm Tris (pH 8.0), 300 mm NaCl, 1 mm dithioerythritol, 1 mm EDTA). The cleaved tags, as well as the His-tagged TEV protease, were separated from the protein of interest via a second nickel-affinity purification. The flow-through was concentrated using centrifugal filters (Amicon Ultra-15, Merck Millipore) and further purified via size exclusion chromatography on a Superdex 200 16/600 prep grade column (GE Healthcare) equilibrated in storage buffer (10 mm Tris (pH 8.0), 150 mm NaCl, 2 mm MgCl_2_). Purified protein was flash frozen in liquid nitrogen and stored at −70 °C until use.

### Spectra and activity assays

Spectra were recorded with proteins in storage buffer at room temperature using a Specord 200 Plus spectrophotometer and a Specord S-300 (Analytik Jena) for experiments with continuous illumination. For light measurements samples were illuminated using a 660-nm light-emitting diode (M660L4 LED, Thorlabs). Dark reversion was monitored at 752 nm. To minimize the influence of the measuring light, neutral density filters were used and the total acquisition time was kept at a minimum.

Activity assays were performed in 0.2-ml reaction tubes in 70-μl reactions containing 20 μm protein (monomer) at various substrate concentrations at 20 °C in reaction buffer (50 mm HEPES (pH 7.0), 150 mm NaCl, 50 mm MgCl_2_). For each substrate concentration at least three time points were measured in triplicate. For light measurements a red LED was used for illumination with 34 milliwatt/cm^2^ (M660L4, Thorlabs), whereas dark measurements were performed in the presence of indirect dim green light. Reactions were stopped by incubation at 95 °C for 1 min and samples were cleared (20,000 × *g*, 5 min) prior to analysis via HPLC UV-visible (UltiMate 3000, Thermo Fisher Scientific) using an adapted method from Enomoto *et al.* (35) as described elsewhere (27).

### Crystallization and refinement

Crystallization experiments were carried out at 20 °C using various protein concentrations with both the full-length and the C terminally-truncated ΔC variants. Crystals from initial hits for the ΔC protein in commercially available screens were optimized in random matrix microseeding experiments. Diffraction quality crystals were grown in drops containing 1 μl of protein at 100 μm and 1 μl of crystallization solution containing seeds (0.1 M bis-Tris (pH 5.5), 1 m ammonium sulfate, 1% (w/v) PEG 3,350) in a sitting drop vapor diffusion setup over a 50-μl reservoir. Crystals were soaked for several minutes in crystallization solution containing 20% (v/v) glycerol before flash freezing them in liquid nitrogen. A complete dataset was collected to a resolution of 2.35 Å at DESY beamline P11 (Hamburg) (36). As the data appeared to be non-merohedrally twinned, the fraction of overlapping reflections was kept to a minimum by optimizing crystal orientation, crystal to detector distance, and small oscillation angles for data collection. Data processing was performed using the XDS package (37). The structure was solved by molecular replacement with an existing model from an earlier low-resolution dataset that was built using partial models from *Dr*BphP (PDB 4O0P) and Cya2 (PDB 2W01), where the PHY domain was rebuilt manually. Structure rebuilding and refinement were carried out with an initial rigid body refinement and torsion angle-simulated annealing, followed by several cycles of manual real space refinement in Coot (38) and maximum-likelihood refinement with Phenix refine (39). Early refinement steps included secondary structure restraints. Five TLS refinement groups per chain were defined based on the TLS Motion Determination server (residues 1–135, 136–317, 318–496, 497–537, and 538–733; corresponding nicely to domains of the PSM (PAS, GAF, PHY), the coiled-coil linker element and the GC domain, respectively) (40, 41) and used throughout the refinement. Cell parameters and statistics of data collection and refinement are provided in Table 1. Figures were generated using PyMOL (42).

**Table 1:** **Summary of data collection and refinement statistics**

**Data collection**	
Space group	C 1 2 1
Cell dimensions	
*a, b, c* (Å)	286.19, 108.82, 67.99
α, β, γ (°)	90.00, 100.84, 90.00
Wavelength (Å)	1.0332
Resolution (Å)	46.847–2.35 (2.45–2.35)*^a^*
*R*_merge_ (%)	8.9 (98.3)
*I*/σ*(I)*	13.78 (1.96)
*CC*_1/2_	0.998 (0.672)
Completeness (%)	96.1 (80.1)
Redundancy	6.77 (6.48)

**Refinement**	
Resolution (Å)	46.847–2.35
No. of reflections	82,007
*R*_work_/*R*_free_	0.1797/0.2269
No. of atoms	
Protein	11,114
Ligand/ion	173
Water	142
*B* factors (Å^2^)	
Wilson	53.0
Protein	68.73
Ligand/ion	70.20
Water	51.59
Root mean square deviations	
Bond lengths (Å)	0.008
Bond angles (°)	0.875
Ramachandran statistics (%)	
Favored/allowed/outliers	98.22/1.71/0.07
PDB entry	6FHT

*^a^* Values in parentheses correspond to the highest resolution shell.

### Hydrogen–deuterium exchange coupled to MS

For hydrogen–deuterium exchange experiments the protein (200 μm) was equilibrated for 1 min either in the dark or under red light illumination (0.7 milliwatt/cm^2^, 630 nm, Luminea LED) before starting the exchange reaction by a 20-fold dilution of the protein in buffer containing D_2_O (10 mm Tris (pD 8.0), 150 mm NaCl, 2 mm MgCl_2_). Aliquots of 5 μl were withdrawn after 10 s, 45 s, 3 min, 15 min, and 60 min, quenched with 50 μl of ice-cold quench buffer (200 mm ammonium formate (pH 2.5)) and immediately flash-frozen in liquid nitrogen. For analysis, the samples were thawed and injected into a cooled HPLC-UHR-TOF system equipped with a pepsin column and a reversed-phase column for peptide separation as described elsewhere (43). Data were analyzed using the Hexicon 2 software package (44).

### C. elegans strains and maintenance

Worms were grown at 20 ºC in constant darkness on agar plates containing nematode growth medium and fed the OP_50_ derivative bacterial strain DA837 (45), which was supplemented with 100 μm biliverdin hydrochloride (Frontier Scientific). The following strains were used: N2 (Bristol), SJU16 = *stjEx9*[P*unc-17*:*IlaC22*:SL2:*dsRed*; P*myo-2:gfp*], SJU17 = *stjEx10*[P*unc-17*:*IlaC22*:SL2:*dsRed*; P*myo-2:gfp*], SJU159 = *stjEx112*[P*unc-17*:*PaaC*:SL2:*dsRed*; P*myo-2:mCherry*] and SJU161 = *stjEx112*[P*unc-17*:*PaaC*:SL2:*dsRed*; P*myo-2:mCherry*].

### Quantification of activity

The WorMotel imaging system was used to quantify locomotor activity in this study (24). Worms were exposed to a brief period of light the day prior to the experiments, when L4 larvae were transferred to fresh plates to stage for first-day adults the following day. On the day of the experiment, polydimethylsiloxane, 24-welled microchips (a gift from Chris Fang-Yen), were filled with molten nematode growth medium-agar and allowed to cool. First-day adults were briefly exposed to light when animals for each strain were transferred to the agar surface of a WorMotel well (6-WT, 6-IlaC22 k27-expressing and 12-PaaC expressing). They were allowed to equilibrate for 1 h after transfer in the dark. At which point animals were exposed to either 5 min of green, red, green and red light, or 300 min of green light. Images were taken every 10 s using a DMK 72BUC02 USB 2.0 monochrome industrial camera. Pixel changes were summed between frames using MatLab software as previously described (24) and then averaged across each time interval. If a worm traveled from one well to another during the time of the recording, then both of those wells were censored.

## Author contributions

S. E. and A. W. data curation; S. E. formal analysis; S. E., R. L., and M. D. N. investigation; S. E. and M. D. N. visualization; S. E., R. L., M. D. N., and A. W. methodology; S. E. writing-original draft; S. E., R. L., M. D. N., and A. W. writing-review and editing; R. L. software; M. D. N. and A. W. resources; A. W. conceptualization; A. W. supervision; A. W. funding acquisition; A. W. validation; A. W. project administration.

## Supplementary Material

Supporting Information

## Footnotes

4 The abbreviations used are:

GC: guanylate cyclase
AC: adenylate cyclase
CTE: cyclase transducer element
PSM: PAS-GAF-PHY photosensory module
PAS: Per/ARNT/Sim domain
GAF: cGMP phosphodiesterase/adenylate cyclase/FhlA domain
PHY: phytochrome-specific domain
CHASE2: cyclases/histidine kinases associated sensory extracellular 2 domain
HDX-MS: hydrogen–deuterium exchange coupled to mass spectrometry
dsRED: *Discosoma sp*. red fluorescent protein
TEV protease: tobacco etch virus protease
LB: lysogeny broth
X-Gal: 5-bromo-4-chloro-3-indolyl-β-d-galactopyranoside
bis-Tris: 2-[bis(2-hydroxyethyl)amino]-2-(hydroxymethyl)propane-1,3-diol
PDB: Protein Data Bank.
