# Supplementary material for: Structure-guided design and functional characterization of an artificial red light–regulated guanylate/adenylate cyclase for optogenetic applications
Source: J Biol Chem. 2018 Apr 25;293(23):9078–89. doi: 10.1074/jbc.RA118.003069 (PMC5995499; doi:10.1074/jbc.RA118.003069)
Supplement: Supporting Information [file supp_293_23_9078__index.html]

Structure-guided design and functional characterization of an artificial red light-regulated guanylate/adenylate cyclase for optogenetic applications — Design and characterization of phytochrome-linked cyclases — Structure-guided design and functional characterization of an artificial red light–regulated guanylate/adenylate cyclase for optogenetic applications — Design and characterization of phytochrome-linked cyclases — Supporting Information 

# Structure-guided design and functional characterization of an artificial red light–regulated guanylate/adenylate cyclase for optogenetic applications

## Supporting Information

- Supporting Information - Figures S1-S5 Table S1 A description of primers used in this work Movie S1 legend PyMol session file illustrating HDX data content description
- Movie S1 - A representative movie showing worm activity during the following pattern of light exposure
- PyMol session file illustrating HDX data - The file HDX\_data.pse contains five objects representing the crystal structure of PaaC&#x0394;C colored according to the difference in relative deuterium incorporation in the dark vs. under red light illumination at the measured time points. Regions with increased deuterium incorporation appear in red, whereas reduced deuteration is shown in blue, according to the scale bar in Figure 5.
