# Supplementary material for: Structure-guided design and functional characterization of an artificial red light–regulated guanylate/adenylate cyclase for optogenetic applications
Source: J Biol Chem. 2018 Apr 25;293(23):9078–89. doi: 10.1074/jbc.RA118.003069 (PMC5995499; doi:10.1074/jbc.RA118.003069)
Supplement: Supporting Information [file supp_RA118.003069_136909_1_supp_124518_p7hglq.pdf]

SUPPORTING INFORMATION

Structure-guided design and functional characterization of an artificial red light-regulated guanylate/adenylate cyclase for optogenetic applications

Stefan Etzl[1], Robert Lindner[2], Matthew D. Nelson[3], and Andreas Winkler[1*]

[1]Institute of Biochemistry, Graz University of Technology, Petersgasse 12/II, 8010 Graz, Austria.
[2]Max Planck Institute for Medical Research, Jahnstrasse 29, Heidelberg, 69120, Germany.
[3]Department of Biology, Saint Joseph's University, Philadelphia, PA 19131, U.S.A.

Running title: *Design and characterization of phytochrome-linked cyclases*

[*]Correspondence to: Andreas.Winkler@TUGraz.at

This file includes:
Figures S1-S5
Table S1
A description of primers used in this work

Movie S1 legend
PyMol session file illustrating HDX data content description

Additional files:
Movie S1
A PyMol session file illustrating HDX data

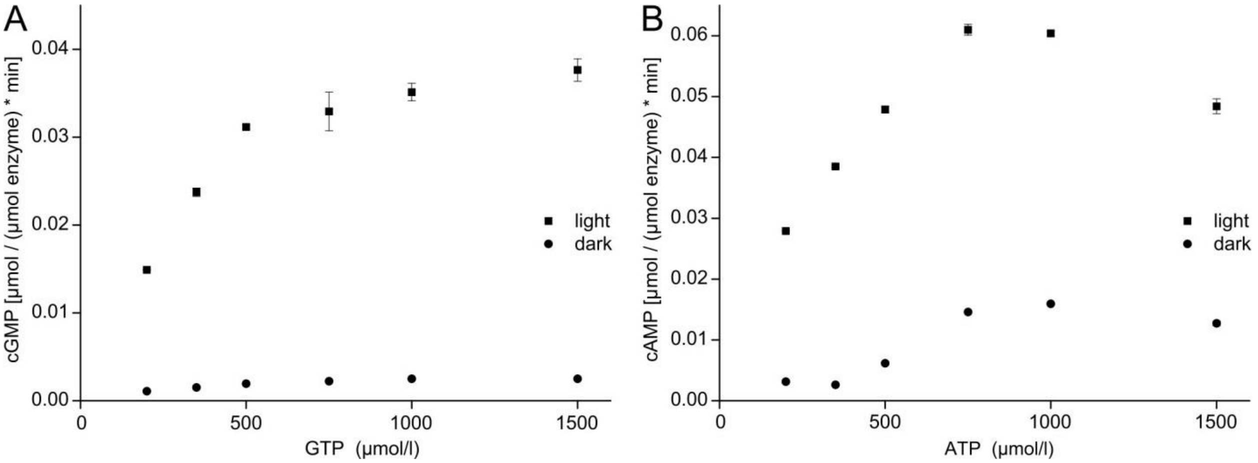

Figure S1: Formation of cyclic nucleotides of phytochrome-linked cyclase variants in dark and red light conditions. **A)** PagC with GTP as a substrate. **B)** PaaC with ATP. Velocities were determined by measurements of cNMP formation for at least three time points where substrate conversion was less than 10%. Product formation was analyzed by HPLC with UV/Vis detection. Error bars represent the error of estimate of linear fits for the initial velocities weighted by the reciprocal of the square of the standard deviation from independent triplicates.

Table S1: Initial velocities of phytochrome-linked cyclase variants and cyclase-only constructs at 1 mM substrate concentration.

	**µmol cNMP/[µmol (enzyme) * min]**	
**Construct + Substrate**	**Light**	**Dark**
PagC + GTP	$(3.5 \pm 0.1) \times 10^{-2}$	$(2.5 \pm 0.1) \times 10^{-3}$
PagC + ATP	$(3.29 \pm 0.12) \times 10^{-4}$	$(1.98 \pm 0.02) \times 10^{-4}$
PaaC + ATP	$(6.04 \pm 0.04) \times 10^{-2}$	$(1.59 \pm 0.02) \times 10^{-2}$
PaaC + GTP	$(4.57 \pm 0.15) \times 10^{-4}$	$(2.90 \pm 0.09) \times 10^{-5}$
Cya2 (403-756) wt + GTP	$(2.36 \pm 0.11) \times 10^{-3}$	
Cya2 (403-756) wt + ATP	$(4.4 \pm 0.1) \times 10^{-5}$	
Cya2 (403-756) E488K + ATP	$(8.1 \pm 0.3) \times 10^{-3}$	
Cya2 (403-756) E488K + GTP	$(4.6 \pm 0.1) \times 10^{-5}$	

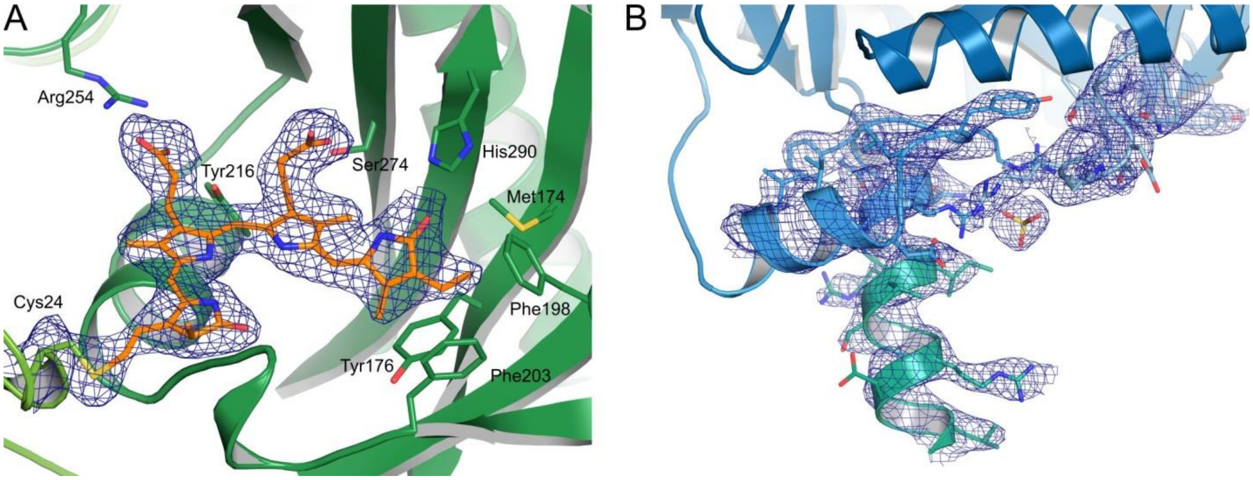

Figure S2: Observed electron density of critical elements. **A)** Biliverdin binding pocket in chain A. Surrounding sidechains are shown in stick representation. The biliverdin chromophore (orange) is covalently attached to Cys24. Electron density is displayed at a contour level of 1 sigma and for clarity residues 256-270 and 450-475 are not shown. **B)** Electron density (1 σ) in the linker-CTE region of chain A and the ^{GC}β4-β5 hairpin of chain B.

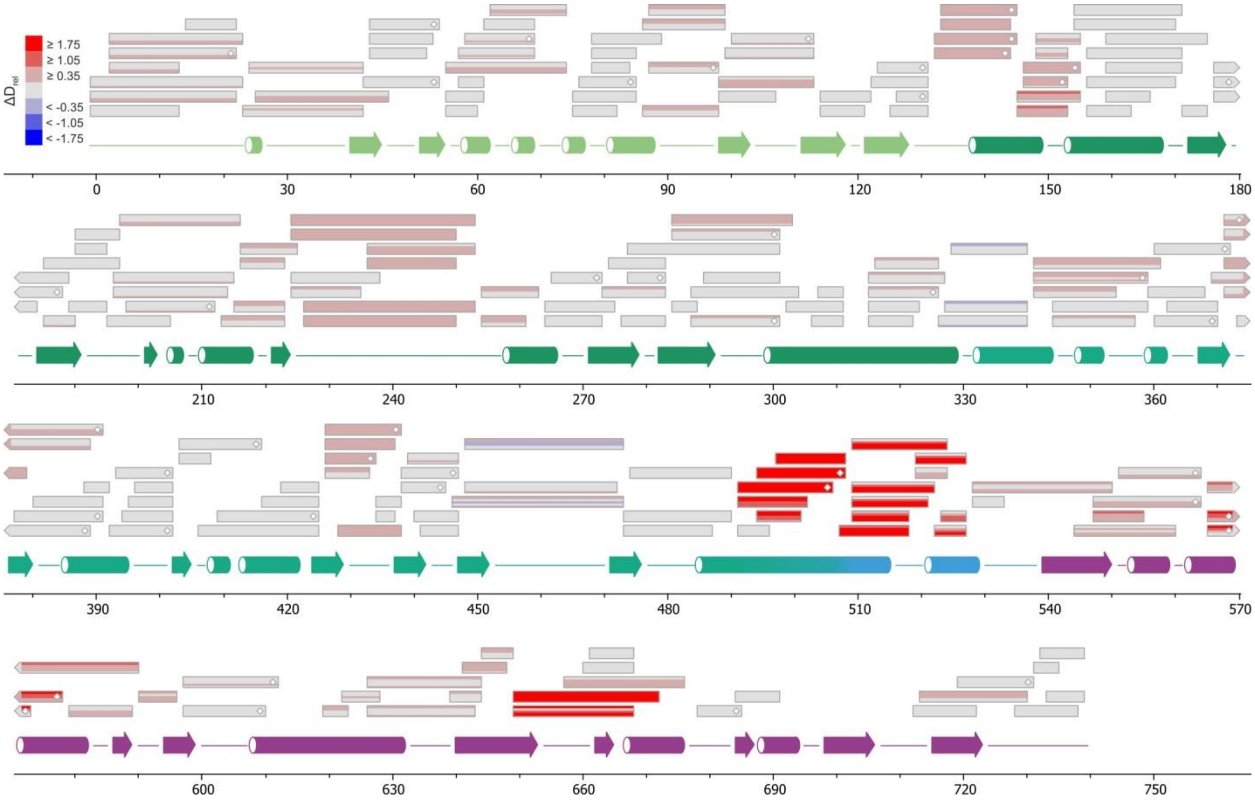

Figure S3: Overview of PagCΔC peptides used for evaluation of HDX-MS data. Each box represents one peptide and consists of five horizontal bars that are colored according to the relative deuteration of red light illuminated PagCΔC compared with dark state PagCΔC. The bars correspond to the measured time points of 10 s, 3 min, 15 min, 45 min and 60 min (bottom to top, respectively). The coloring scheme is according to the scale bar at the top left. Peptides marked with a diamond are MS^2 confirmed. The secondary structure elements are colored according to the scheme in Fig. 1A.

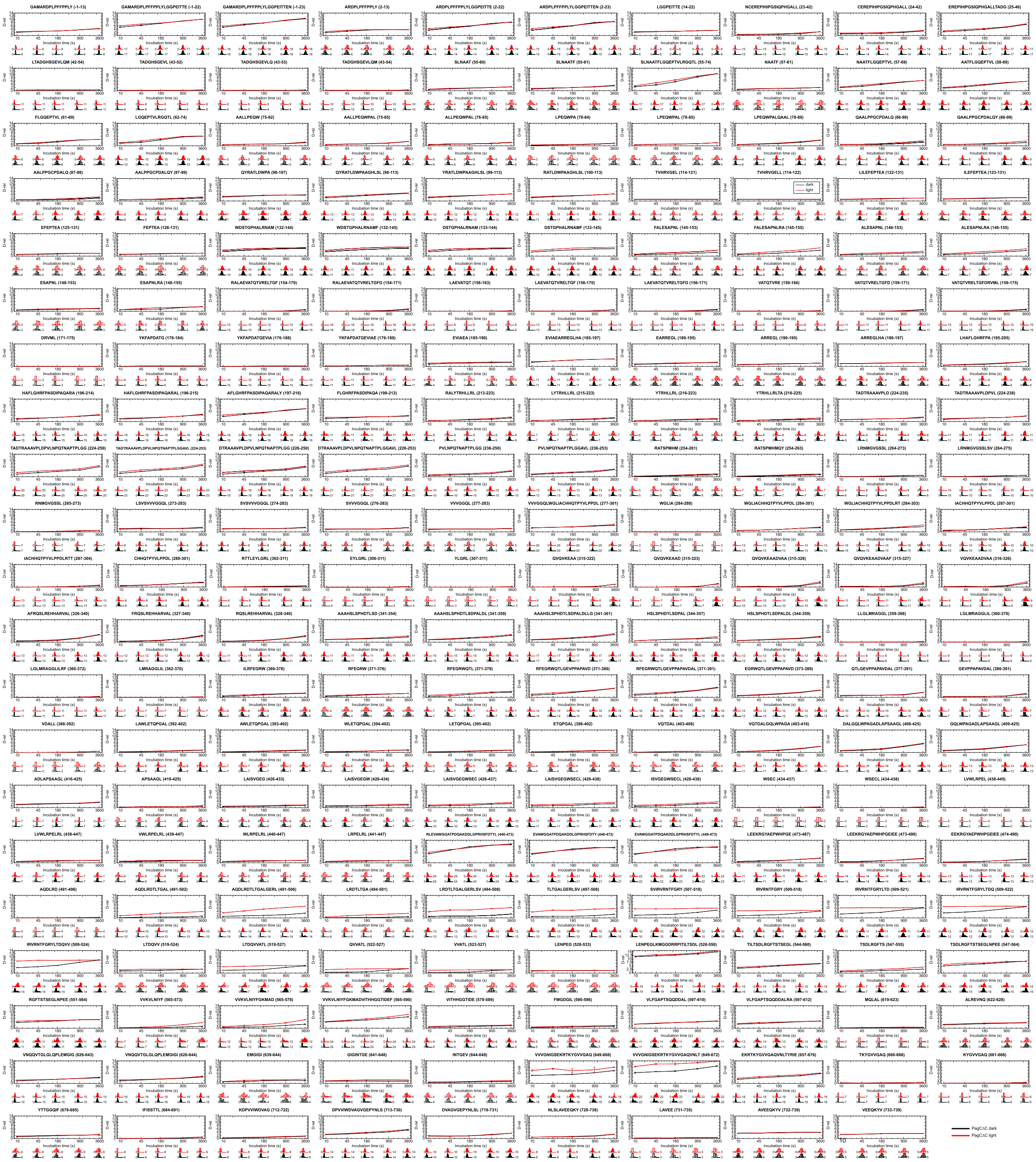

Figure S4: Relative deuterium incorporation of all evaluated peptides. The plots show the relative deuterium uptake D-rel ($D_{deuterated}$- $D_{undeuterated}$) in the dark (black) and under red light (red) for various deuteration times. Error bars indicate the standard deviation of three independent measurements. On top of the plots the sequences of the peptides are shown with the absolute numbering in parentheses. The deuterium distributions below the plots depict the abundance of deuterated species, ranging from undeuterated to fully deuterated backbone amides of the respective peptide.

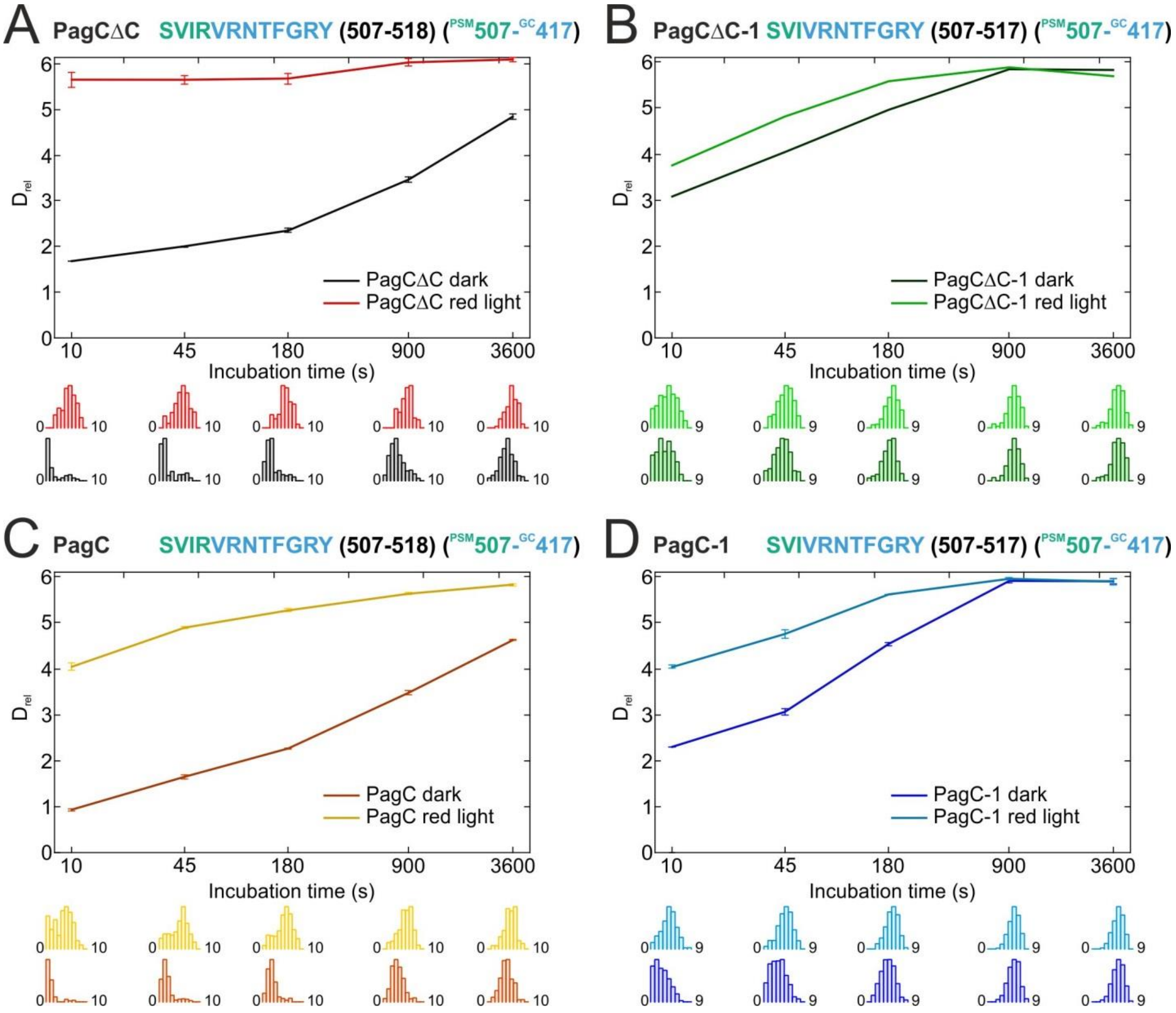

Figure S5: Comparison of representative peptides in the linker-CTE region of various constructs. The plots show relative deuterium incorporation (D_{rel}) in the dark state protein and under red light illumination for various time points. **A)** PagCΔC is destabilized in the linker region upon red light illumination (black = dark, red = illuminated). **B)** Despite showing inverse cyclase regulation, the corresponding peptide in PagCΔC-1 is also destabilized under red light (green = dark, light green = illuminated)*. **C)** Full-length PagC (including the C-terminal extension of the cyclase core) has a similar behavior as the truncated ΔC construct (brown = dark, yellow = illuminated). **D)** Full-length PagC-1 is as well destabilized in the linker upon illumination (blue = dark, cyan = illuminated). The peptide sequences with absolute and component-specific numbering are shown above the plots. Error bars represent the standard deviation of three independent measurements. The plots below the graphs depict abundance distributions of all measured species from undeuterated to fully exchanged. *For PagCΔC-1 only single measurements were performed.

Primers used in this work

The following constructs were created in PCR reactions by deletions of the initial construct PaaC+2 in the pET M11 vector using the universal forward primer 5'-GTTCGTAATACCTTTGGTCGTTATCTGACCGATCAGGTTGTTGC and the below reverse primers:

PaaC-4: 5'-GACCAAAGGTATTACGAACCAGACGTTCACCCAGTGCAC
PaaC-3: 5'-GACCAAAGGTATTACGAACGCTCAGACGTTCACCCAGTG
PaaC-2: 5'-GACCAAAGGTATTACGAACAACGCTCAGACGTTCACCC
PaaC-1: 5'-GACCAAAGGTATTACGAACAATAACGCTCAGACGTTCACCCAG
PaaC: 5'-GACCAAAGGTATTACGAACACGAATAACGCTCAGACGTTCACC
PaaC+1: 5'-GACCAAAGGTATTACGAACATCACGAATAACGCTCAGACGTTC

Constructs PaaC+3 - PaaC+10 were created by insertions in template PaaC+2 using the universal reverse primer 5'-CAGATCACGAATAACGCTCAGACGTTCACCCAGTGCAC with the following forward primers:

PaaC+3: 5'-AGCGTTATTCGTGATCTGAATGTTCGTAATACCTTTGGTCGTTATCTGAC
PaaC+4: 5'-AGCGTTATTCGTGATCTGAATCGTGTTCGTAATACCTTTGGTCGTTATCTGAC
PaaC+5: 5'-AGCGTTATTCGTGATCTGAATCGTGCAGTTCGTAATACCTTTGGTCGTTATCTGAC
PaaC+6: 5'-AGCGTTATTCGTGATCTGAATCGTGCACTGGTTCGTAATACCTTTGGTCGTTATCTGAC
PaaC+7: 5'-AGCGTTATTCGTGATCTGAATCGTGCACTGACCGTTCGTAATACCTTTGGTCGTTATCTGAC
PaaC+8: 5'-AGCGTTATTCGTGATCTGAATCGTGCACTGACCCAGGTTCGTAATACCTTTGGTCGTTATCTGAC
PaaC+9: 5'-AGCGTTATTCGTGATCTGAATCGTGCACTGACCCAGTCTGTTCGTAATACCTTTGGTCGTTATCTGAC
PaaC+10: 5'-AGCGTTATTCGTGATCTGAATCGTGCACTGACCCAGTCTAATGTTCGTAATACCTTTGGTCGTTATCTGAC

For the reversion of the ^{GC}E488K mutation to the corresponding PagC variants the following primers were used:

^{GC}K488E_fw: 5'-ACCATTGATGAATTCATGGGTGATGGTATTCTGGTGCTG
^{GC}K488E_rv: 5'-CATGAATTCATCAATGGTGCCACCATGATGGGTAATAAC

Constructs for *C. elegans* expression were made using the following primers:

Punc-17:IlaC22:SL2:dsRED

oSJU30_fw: 5'CACCAATCATTTCTCCCCTCC (Binds 5' of *unc-17*)
oSJU31_fw: 5'CATGACAAAGTGGTGACACTGG (Nested to oSJU30)
oSJU36_rv: 5'TAGTCATAAGGCATCCGCGGGCCATCTTGTTTTAGTAGGTTACTATTTTGAAC (Binds to *unc-17* and adds IlaC22 complementary sequence)
oSJU37_fw: 5'TTGTTCAAAATAGTAACCTACTAAAACAAGATGGCCCGCGGATGCCTTATG (Binds to IlaC22 and adds *unc-17* complementary sequence)
oSJU10_rv: 5'TGGGTCCTTTGGCCAATCCCGGCCGCTTAGTGGTGGTGGTGATGATG (Binds to IlaC22 and adds operon complementary sequence)
oSJU11_fw: 5' AAGCATCATCACCACCACCACTAAGCGGCCGGGATTGGCCAAAGG

(Binds to operon and adds IlaC22 complementary sequence)
oSJU349_rv: 5'GGAAGCGAGAAGAATCATAATG (Binds 3' of the dsRED sequence)
oSJU350_rv: 5'GAGAAGAATCATAATGGGGAAG (Nested to 0SJU349)

Primer pairs: oSJU30/oSJU36; oSJU37/oSJU10; oSJU11/oSJU349; oSJU31;oSJU350

Punc-17:PaaC:SL2:dsRED

oSJU322_rv: 5'AAGAATGGAAGTGGATCTCGAGCCATCTTGTTTTAGTAGGTTACTATTTTG
(Binds to *unc-17* and adds PaaC complementary sequence)
oSJU323_fw: 5'CAAAATAGTAACCTACTAAAACAAGATGGCTCGAGATCCACTTCCATT
(Binds to PaaC and adds *unc-17* complementary sequence)
0SJU286_rv: 5'TGGGTCCTTTGGCCAATCCCGGCCGCTTAATGGTGGTGATGGTGATGAG
(Binds to PaaC and adds operon complementary sequence)
0SJU287_fw: 5'TAGTGCTCATCACCATCACCACCATTAAGCGGCCGGGATTGGCCAAAG
(Binds to operon and adds PaaC complementary sequence)

Primer pairs: oSJU30/oSJU322; oSJU323/oSJU286; oSJU287/oSJU349; oSJU31;oSJU350

Movie S1: A representative movie showing worm activity during the following pattern of light exposure: Green - Red - Green - Red (5 minutes each). The top two rows (1 and 2) contain PaaC-expressing animals; row 3 contains IlaC22 k27-expressing animals; and row 4 contains WT animals. The images were taken at 10-minute intervals and the video speed has been increased 4-fold.

PyMol session file illustrating HDX data: The file HDX_data.pse contains five objects representing the crystal structure of PaaCΔC colored according to the difference in relative deuterium incorporation in the dark vs. under red light illumination at the measured time points. Regions with increased deuterium incorporation appear in red, whereas reduced deuteration is shown in blue, according to the scale bar in Figure 5.
